# Promoting health literacy during Covid-19 pandemic–valuable partnership with Portuguese pharmacists: Filipa Malcata

**DOI:** 10.1093/eurpub/ckac131.325

**Published:** 2022-10-25

**Authors:** F Malcata, B Raposo, D Costa, MT de Arriaga

**Affiliations:** 1 Public Health Unit, ACES Porto Ocidental, Porto, Portugal; 2 Directorate-General of Health, Lisbon, Portugal

## Abstract

**Issue/Problem:**

COVID-19 pandemic has amplified the amount of health information available; however, hard-to-reach populations remain and much of the information available is not accurate. Therefore, the use of new proximity approaches in the promotion of health literacy is essential because people face challenges and barriers when trying to find information relevant to them.

**Description of the problem:**

From the beginning of the COVID-19 pandemic in 2020, the Division of Literacy, Health and Wellbeing of the Directorate-General of Health in Portugal developed Social Mobilization and Community Engagement training sessions-which included a presentation and a toolkit of flyers/posters/videos. These sessions had the goal of disseminating public health measures through good practices in health literacy and communication methods/skills to promote efficient communication among people who play an active role (as microinfluencers) in prevention and mitigation of risks associated with COVID-19, as is the case of pharmacists.

**Results:**

A total of 200 Portuguese pharmacists enrolled on the Social mobilization training session in November 2021. More than 150 initiatives were implemented thereafter by those microinfluencers (pharmacists) in the local communities-31% directed to children or young people, 31% to older adults, and 13.8% to migrants or tourists. 70% of the participants considered the materials of the toolkit as adequate and sufficient, and 62.1% answered that people in the community showed interest for those materials available in the pharmacies.

**Lessons:**

This Social mobilization project was important because it assembled and preserved trust- basing the information of the training on reliable sources about COVID-19 virus. It also trained the microinfluencers (pharmacists) in communication strategies. All these approaches were important to prevent and mitigate the impact of COVID-19 via adoption of non-pharmacological measures and promotion of vaccination.

**Key messages:**

• Portugal was pioneer in promoting Social mobilization and community engagement training sessions, maintaining a proximity communication, tailoring the information, and maximizing its impact.

• Within the group of health professionals, pharmacists are a privileged group to promote these communication strategies and social mobilization due to their proximity with the local community.

